# Reversible cerebral vasoconstriction syndrome (RCVS): an interesting case report

**DOI:** 10.1186/s10194-021-01225-7

**Published:** 2021-04-06

**Authors:** Mansoureh Togha, Mahsa Babaei, Parvin Ganji Ghelichi

**Affiliations:** 1 Neurology ward, Sina Hospital, School of Medicine, Tehran University of Medical Sciences, Tehran, Iran; 2 Headache department, Irainian Center of Neurological Researches, Institute of Neuroscience, Tehran University of Medical Sciences, Tehran, Iran; 3 Department of Medicine, Shahid Beheshti University of Medical Sciences, Tehran, Iran; 4 Department of medicine, Division of obstetrics & Gynecology, Milad hospital, Tehran, Iran

**Keywords:** Pheochromocytoma, Reversible cerebral vasoconstriction syndrome, Thunder-clap headache

## Abstract

**Background:**

Reversible Cerebral Vasoconstriction Syndrome is a condition of transient cerebral vascular spasms, which usually presents with recurrent thunderclap headaches and recovers within 3 months. Several probable triggers and underlying factors, such as sex hormones, vasoactive drugs, head trauma or surgery, and tumors, have been implicated.

**Case presentation:**

In this paper, we present **a** 53-year-old woman with thunderclap headaches and normal lab tests who was radio-clinically diagnosed with reversible cerebral vasoconstriction syndrome and treated accordingly. Then, she experienced the recurrence of RCVS after about 2 years and headaches after 1 year in association with high blood pressure, high blood sugar, hypothyroidism, hyperlipidemia, and a urine metanephrine level of 5 times higher than the normal limit, suggesting a diagnosis of pheochromocytoma. After confirmation of the diagnosis with further imaging studies, surgical removal of the tumor resolved all the signs and symptoms.

**Conclusion:**

Often underdiagnosed, pheochromocytoma could be an important condition associated with RCVS. It is important for clinicians to bear this diagnosis in mind while dealing with similar cases of recurrent thunderclap headaches.

## Introduction

Thunderclap headache (TCH) is defined as a high-intensity headache with an abrupt onset, reaching its peak intensity within less than one minute and lasting for more than five minutes. This type of headache is usually indicative of serious underlying causes and is rarely a primary disorder [1].

Reversible Cerebral Vasoconstriction Syndrome (RCVS) is a condition of vascular spasms in the brain, mainly presenting with a recurrent, severe, diffuse type of TCH that may be present or recurrent for one or 2 weeks from its onset and usually resolves within 3 months. This is a poorly understood condition, which can be associated with focal neurological deficits and seizures. The “string of beads” appearance on angiography, consisting of segmental vasodilations and vasoconstrictions, is the radiological characteristic of RCVS [1]. RCVS [1, 2] and Subarachnoid Hemorrhage (SAH) [2] are the most common causes of TCH, making it a medical emergency requiring immediate consideration [1, 2].

Several triggers and underlying factors contribute to formation of RCVS and its final management is based on the recognition and resolution of the underlying cause and avoidance of probable triggers [3–6]. Accordingly, catecholamine secreting tumors, specifically pheochromocytomas, have been reported as rare but important causes of RCVS [3, 7].

Pheochromocytoma is a tumor of chromaffin cell that develops in the adrenal medulla. Manifestations of functional pheochromocytomas are mainly due to excessive amounts of catecholamine release [8]. A diagnosis is usually made based on clinical manifestations and lab tests and the final management usually includes surgical removal of the tumor [8–12].

We present a case of TCH with a diagnosis of recurrent RCVS associated with pheochromocytoma. This case posed a diagnostic challenge and took 3 years to reach a final diagnosis and identify the underlying cause of thunderclap headache, which was pheochromocytoma. Interestingly, all signs and symptoms resolved after tumor resection.

## Case presentation

About 6 years ago, a 53-year-old woman who was an expert gynecologist presented with severe headaches for which she was admitted to the neurology ward of the hospital where she was working. She complained about three episodes of severe, sudden-onset headaches in the last 2 weeks. Each headache episode started spontaneously, reached its maximum intensity within 1 min, and lasted for 5 to 10 min. The headache was pulsatile starting from the occipital area extending to the frontal region. She also complained about less severe headaches with a shorter duration in the next days, mainly triggered by the Valsalva maneuver. The pain was not positional and did not respond to any analgesics or opioids.

On admission, her physical and neurological examinations including fundoscopic evaluation were normal. Brain Magnetic Resonance Imaging (MRI) with and without contrast, Magnetic Resonance Arteriography (MRA) and Magnetic Resonance Venography (MRV) were unremarkable.

A 24-h Ambulatory Blood Pressure Monitor (ABPM) recorded a mean blood pressure of 110/70 that reached its peak at 180/120 during her headache attacks. The heart rate was normal.

Hematological tests and 24-h urine vanillylmandelic acid (VMA) were all normal. Abdominopelvic sonography also reported normal findings. The patient did not consent to a lumbar puncture.

Because of repeated attacks of thunderclap headache and ruling out SAH and other possible causes of TCH, a diagnosis of RCVS was made and IV magnesium and oral verapamil were initiated with careful cardiac monitoring. TCH did not repeat and other milder headache attacks significantly improved and resolved thereafter. The patient was discharged from the hospital with oral verapamil and magnesium. She complained about dizziness and presyncope attacks for 1 month after discharge that resolved spontaneously. Her medications were tapered off gradually and discontinued.

After 2 years of being asymptomatic, she started to experience tachycardia and tachypnea while lying down from a sitting position. She felt cold in her head and developed facial pallor or flashing after experiencing anger, stress, tiredness, or hot temperature. She also experienced dizziness on standing occasionally. One month later, she repeatedly experienced TCH-like attacks. Her maximum blood pressure was 200/140 occasionally at the time of the headache attacks and was normal in between. She underwent physical and neurologic exams, blood and urine sample tests, ABPM, ECG, IOP measurement, trans-Cranial Color Doppler (TCCD), and brain MRI, MRA, and MRV. There was a small hematoma in the right parietal lobe without edema and compression effect that was reported as a hemorrhagic infarct. In brain MRA, segmental arterial spasms were noted and increased velocity of Right Middle Cerebral Artery (RMCA), suggestive of vasospasm, was reported on TCCD. All other test results including those related to collagen vascular diseases were normal.

With a diagnosis of recurrent RCVS, which is a rare condition, oral administration of magnesium 600 mg/day and verapamil 200 mg/day was initiated. Medications improved her signs and symptoms but were not able to resolve them completely. Verapamil was tapered to a dose of 80 mg/day orally within 4 months. In addition, magnesium administration was continued at a dose of 400 mg/d. Two questions were unanswered regarding the patient’s diagnosis: the repeated RCVS attacks, which is quite unusual, and the continuous need for verapamil and magnesium. Two years later, despite receiving the medications, she experienced recurrence of pulsatile headache attacks of short duration (about 10 min) that were less severe than previous bouts. Sedatives were prescribed and she was advised to rest, avoid stress, and sleep well, but none of them could relive the headaches.

Unexpectedly, after 6 months, she developed hypertension and her lab tests revealed high blood sugar. She also developed general skin pruritus and unusual pustules. Further investigations revealed that the urinary metanephrine level was 5 times higher than the normal range, indicated a diagnosis of pheochromocytoma. Additionally, Computerized Tomography (CT) scan revealed a mass in the right adrenal gland. Phenoxybenzamine was initiated to control and reduce her blood pressure in order to prepare the patient for surgery. Fourteen days later, laparoscopic hemi-adrenalectomy was performed, and verapamil was discontinued after the surgery. Thereafter, all the manifestations, including headache attacks and hypertension, resolved completely and all the routine hematological and urine tests, especially blood sugar became normal.

## Discussion

RCVS mostly affects middle aged women [13] and is believed to be more prevalent than what is reported in the literature [4]. Therefore, it is commonly underdiagnosed [4, 14, 15]. Several precipitating factors including exertion [1, 3], coughing [3], sexual activity [1, 3], bathing [3], and Valsalva maneuver [1], a number of underlying causes such as pregnancy related conditions [3, 7], postpartum [1, 3, 4, 7], exposure to vasoactive drugs [1, 3, 4, 7], use of licit or illicit drugs [1, 4, 7], catecholamine excess [3, 4, 7], head and neck related disorders including trauma, head and neck surgery, carotid endarterectomy, and cervical artery dissection [3, 7], and various medical conditions such as exposure to immunosuppressant drugs or blood products [3, 7] have been implicated in RCVS.

As described earlier in the introduction section, RCVS mainly presents with TCH-like headaches [1]. Besides, any new TCH is a medical emergency prompting further investigations [1, 2, 16], including careful physical and neurologic examination, a brain CT scan without contrast as soon as possible, and a lumbar puncture in case of an inconclusive CT scan to evaluate SAH. After exclusion of SAH, further evaluations are needed to find the underlying cause [2, 17, 18], because according to ICDH3, there is poor evidence suggesting that TCH is a primary disorder. Therefore, primary TCH is a diagnosis of last resort and should be mentioned after satisfactory exclusion of organic underlying causes, mainly with normal brain imaging as well as observation of normal brain vessels and normal Cerebrospinal Fluid (CSF) [1].

Diagnosis of RCVS is based on clinical manifestations and radiological findings including the “string of beads” appearance on angiography [1]. Lab tests are usually normal in RCVS [2]. Similarly, radiologic studies can be normal in a number of cases [19], especially during the first week of symptoms. However, any new headache fulfilling the criteria of RCVS with normal imaging and lab test results can be regarded as an “Acute Headache Probably Attributed to RCVS” [1]. If the clinical and radiological findings are inconclusive or the underlying cause is still missing, routine blood tests, urinalysis, urine vanillylmandelic acid and 5-hydroxyindoleacetic acid levels, serum and urine toxicology screens, ESR, CRP, infectious and rheumatological panel tests, and CSF examination are indicated [5].

Repeated headache attacks can be triggered by the Valsalva maneuver or exertion in RCVS [20]. These factors also triggered headache attacks in our patient. Therefore, such headache episodes do not preclude RCVS.

Management of RCVS includes treatment of the underlying cause(s) and avoidance of triggers and precipitants. Calcium channel blockers such as nimodipine and verapamil are used to relieve the headache and acute symptoms. They can also prevent further problems [3–6].

Headache may persist after the resolution of RCVS signs and symptoms; however, there are limited studies in this regard and the headache frequency and type are not well defined yet. Our patient had mild persistent headaches after the second bout of RCVS.

Complications of RCVS include ischemic stroke and intracranial hemorrhages [3, 4, 14]. However, the long-term outcome of RCVS is usually excellent [5, 13, 14]. A number of studies have reported a low risk of recurrence for RCVS in the long term [20], which is usually due to re-exposure to RCVS triggers [5, 15, 21]. Nonetheless, some authors believe that the risk of recurrence is underestimated. In case of RCVS recurrence, a comprehensive evaluation is necessary for RCVS, as explained earlier in detail, to find the underlying cause for appropriate management [15].

Vasoactive substances and catecholamine excess have been categorized as the most important precipitants of RCVS [3, 7], and diagnostic evaluation of RCVS includes these entities [5]. In our case, recurrence of thunderclap headaches after 3 years and repeated investigations suggested that pheochromocytoma was the underlying cause of RCVS, and the signs and symptoms completely resolved with proper management of pheochromocytoma.

According to the literature and our case, functional pheochromocytomas have diverse manifestations, such as hypertension, orthostatic hypotension, hyperhidrosis, palpitation, tachycardia, facial pallor and rarely flushing, fever, anxiety and panic attacks, severe constipation, etc., which are caused by excessive amounts of catecholamine release [8, 22, 23]. It is important for clinicians to keep pheochromocytoma in mind as a differential diagnosis for such diverse manifestations. Measurement of 24-h urine vanillylmandelic acid is the most sensitive initial test for diagnosis of pheochromocytoma [24]. CT scan and MRI are used for localization and further assessment of tumor characteristics. Further investigations such as metaiodobenzylguanidine (MIBG) scintigraphy might be used depending on the case. The best cure for pheochromocytomas, especially functional and non-metastasized ones, is to remove them surgically [8, 11, 12].

Patients should receive α1-blockers such as phenoxybezamine for 10–14 days prior to surgery to avoid hypertensive crisis. Calcium channel blockers and β-blockers can be used as alternatives or adjunct treatments to prevent tachycardia and disrhythmias. In our case, verapamil and phenoxybenzamine were administered prior to surgery. Laboratory tests (1 month later, 6 months later, 1 year later, and then annually) and imaging studies (1 year after the operation) are necessary postoperatively [11, 12]. About 10% of pheochromocytomas metastasize to other organs requiring different, case-based diagnostic and therapeutic approaches [8].

Pre-syncope attacks were associated with pheochromocytoma in our patient, which has been reported in previous studies. Some studies have attributed it to the effects of dopamine produced by some pheochromocytomas and paragangliomas [25–27]. Another study attributed hypotension and syncope in pheochromocytoma to excess epinephrine secretion and volume depletion [28]. Accordingly, several studies have reported that pre-syncope attacks might occur because of transient autonomic dysregulations caused by intermittent excessive secretion of catecholamines [29] or due to altered sympathetic vascular modulation [30]. Another suggested hypothesis is that chronic norepinephrine secretion from phaochromocytoma downregulates the vascular adrenergic receptors causing orthostatic hypotension. Then, it results in reflex stimulation of central sympathetic discharge and causes a further rise in plasma norepinephrine, which aggravates the adrenergic receptor downregulation [31].

Another interesting finding in our patient, in line with previous case reports, was the association of intracranial hemorrhages and ischemic events with pheochromocytoma [32–34].

To the best of our knowledge, three cases of TCH associated with pheochromocytoma [35–37], and four cases of RCVS accompanied by pheochromocytoma [35, 38–40] have been reported in the literature. This emphasizes the need of further investigation of the relationship of RCVS and TCH with pheochromocytoma and excessive catecholamine production indicating the importance of including pheochromocytoma in the differential diagnosis of the underlying causes of RCVS or thunderclap headache, especially in case of recurrence, even if the initial investigations are unremarkable.

## Conclusion

Pheocromocytoma has many different manifestations. As stated in this paper and in line with other previous studies, RCVS is a growing manifestation of pheocromocytoma and clinicians should evaluate patients presenting with RCVS for the possibility of an underlying pheochromocytoma.

## Data Availability

Data is available with corresponding author upon request.
